# Hsa_circ_0046523 Mediates an Immunosuppressive Tumor Microenvironment by Regulating MiR-148a-3p/PD-L1 Axis in Pancreatic Cancer

**DOI:** 10.3389/fonc.2022.877376

**Published:** 2022-05-30

**Authors:** Xiaowei Fu, Gen Sun, Shuju Tu, Kang Fang, Yuanpeng Xiong, Yi Tu, Ming Zha, Tao Xiao, Weidong Xiao

**Affiliations:** ^1^ Department of General Surgery, The First Affiliated Hospital of Nanchang University, Nanchang, China; ^2^ Department of Pathology, The First Affiliated Hospital of Nanchang University, Nanchang, China; ^3^ Institute of Digestive Surgery, Nanchang University, Nanchang, China

**Keywords:** hsa_circ_0046523, miR-148a-3p, PD-L1, tumor microenvironment, pancreatic cancer

## Abstract

**Background:**

Circular RNAs (circRNAs) are a novel type of non-coding RNA, play an important role in the progression of tumors. However, the function and mechanism of circRNAs in regulating immune microenvironment of pancreatic cancer (PC) remain largely unclear.

**Methods:**

The effects of hsa_circ_0046523 expression on proliferation, migration and invasion of PC cells were analyzed by CCK8 and Transwell assays. Flow cytometry was used to detect the proportion of CD4^+^ T cells, CD8^+^ T cells and Tregs in peripheral blood mononuclear cells (PBMCs) after co-culture, and the apoptosis, depletion and function of CD8^+^ T cells. The expression levels of immunoregulatory cytokines were detected by enzyme linked immunosorbent assay (ELISA). The dual-luciferase reporter was performed to determine the interaction between hsa_circ_0046523, miR-148a-3p, and PD-L1. Rescue experiments and PD-L1 blocking experiments were employed to investigate whether hsa_circ_0046523 exerts its biological function by miR-148a-3p/PD-L1 in PC. Furthermore, an immunocompetent murine PC model was established to confirm these findings.

**Results:**

Hsa_circ_0046523 expression was remarkably upregulated in PC tissues and cell lines. Moreover, high expression of hsa_circ_0046523 was correlated with advanced pathological stage and poorer prognosis. Hsa_circ_0046523 overexpression promoted the proliferation, migration and invasion of PC cells *in vitro*. Co-culture experiments confirmed that forced expression of hsa_circ_0046523 could decrease the proportion of CD4^+^ and CD8^+^ T cells, as well as increase the proportion of Tregs among peripheral blood mononuclear cells (PBMCs). Meanwhile, hsa_circ_0046523 overexpression promoted the apoptosis and exhaustion of CD8^+^ T cells, inhibited CD8^+^ T cell function, increased the secretion of immunosuppressive cytokines IL-10 and TGF-β, and decreased the secretion of immune effector cytokines IFN-γ and IL-2 among PBMCs. Mechanistically, hsa_circ_0046523 exerted its biological function by binding to miR-148a-3p to upregulate PD-L1 expression in PC. Moreover, these immune modulating functions of miR-148a-3p/PD-L1 axis were also confirmed in an immunocompetent murine PC model.

**Conclusions:**

Our study suggests that hsa_circ_0046523/miR-148a-3p/PD-L1 regulatory axis mediates PC immunosuppressive microenvironment and these molecules are expected to be new targets for remodeling tumor immune microenvironment of PC.

## Introduction

Pancreatic cancer (PC) is a highly aggressive malignant tumor of the digestive system. Its incidence and mortality are annually rising over the worldwide, and it is expected that PC will become the second most common cause of death among malignancies in 2030 (1). Currently, the treatment of PC mainly includes surgery, radiotherapy and chemotherapy. Surgery represents the only potentially curative approach for resectable and partial borderline resectable or local advanced PC with well response to neoadjuvant therapy. Nevertheless, the high recurrence rate even in patients who have undergone curative resection is still a major issue in the treatment of PC. Despite radical resection combined with adjuvant chemotherapy has improved the prognosis of PC patients to a certain extent, the overall 5-year survival rate of PC is only approximately 10% (2). Therefore, it is of great importance to elucidate the underlying molecular mechanisms of carcinogenesis and progression of PC.

Tumor microenvironment (TME) commonly refers to the internal environment of tumor occurrence and development, which containing tumor cells, immune cells, fibroblasts, endothelial cells, bone marrow-derived inflammatory cells, signaling molecules and a surrounding extracellular matrix (ECM) (3, 4). The immune compositions among TME was named as tumor immune microenvironment (TIME), which included various immune cells, stromal cells, tumor cells and immune cytokines. TIME is a key regulator of carcinogenesis that controls the sequence of tumor development and progression, as well as the tumor response to immunotherapy (5). The adaptive cellular immune response dominated by T cells represents the main mechanism of tumor cell clearance within the TIME. Effector T cells are primarily involved in this process, including CD4^+^ T cells and CD8^+^ T cells. Upon activation, CD4^+^ T cells could differentiate into CD4^+^ helper T cells, secrete IL-2 to activate CD8^+^ cytotoxic lymphocytes (CTLs), which take essential roles for killing tumor cells (6, 7). However, CTLs encounter dysfunction and exhaustion due to immune-related tolerance and immunosuppression within the TIME during cancer progression. PD‐L1 is a checkpoint receptor that can be targeted for relieving exhaustion of CD8^+^ T cells and thereby eliminating antigen‐expressing cancer cells (8). Recent studies have shown that tumor cells and the TME could upregulate PD-L1 expression, activate the PD-1/PD-L1 signaling pathway, inhibit T cell activation and proliferation, and induce T-effector cell apoptosis (9–11). However, the expression of PD-L1 in PC and its regulatory mechanism have not been fully clarified.

MicroRNAs (miRNAs) are a type of single-stranded RNA that are 19 to 23nt in length, which can negatively regulate their target gene expression by binding to the 3′-untranslated region (3′-UTR) of target mRNAs (12). As a member of the miR-148/152 family, the miR-148a-3p gene is located at chromosome 7p15.2. MiR-148a has been reported to downregulate and exert a tumor suppressor in various types of digestive system cancer, including hepatocellular carcinoma (13), cholangiocarcinoma (14), and gastrointestinal cancer (15, 16). Our previous studies revealed that miR-148a-3p expression was significantly reduced in PC tissues, and closely related to tumor size, histological grade, lymph node metastasis, TNM staging and prognosis. Moreover, miR-148a-3p upregulation has been found to inhibit epithelial-mesenchymal transition (EMT) and stem transformation of PC cells through the Wnt1/10b-mediated Wnt/β-catenin signaling pathway (17, 18). In DNA mismatch repair-deficient colorectal cancer, miR-148a-3p negatively regulates tumor cell PD-L1 expression and decreased levels of miR-148a-3p contributes to the immunosuppressive microenvironment (19). Based on GEPIA database (http://gepia.cancer-pku.cn/), PD-L1 was found to be highly expressed in PC tissues. miRNA target prediction databases PicTar, TargetScan and miRanda indicated PD-L1 possessed an miR-148a-3p targeting binding sequence. Therefore, it is reasonable to speculate that miR-148a-3p/PD-L1 axis take part in regulating the TIME of PC.

Circular RNAs (circRNAs) are a subclass of endogenous non-coding RNA (ncRNA) that are formed by exon skipping or back-splicing events (20). CircRNAs can function as miRNA sponges, RNA binding protein (RBP) sponges, and regulators of transcription, and few circRNAs can be translated into proteins/peptides (21). As the competitive endogenous RNAs (ceRNAs), circRNAs can compete for miRNA-binding sites and affect miRNA activities. Emerging evidence shows that circRNAs possess closely associated with the pathogenesis and progress of various cancers. Previous studies have demonstrated that a number of circRNAs were aberrantly expressed in PC (22–24). Nevertheless, the role and mechanism of circRNAs in regulating immune microenvironment of PC remain to be elucidated.

In the present study, aberrantly upregulated circRNAs with miR-148a-3p binding sites in PC were screened out through bioinformatics analysis, and hsa_circ_0046523 was chosen to further function experiments. More importantly, we firstly attempted to reveal the exist of hsa_circ_0046523/miR-148a/PD-L1 regulatory axis and its effect on the TIME of PC. It would provide new insights for the upstream and downstream regulatory mechanism of miR-148a-3p in PC, as well as provide new potential targets for remodeling TIME of PC in future.

## Materials and Methods

### Patient Samples

Fifty-seven pairs of pancreatic cancer tissues and corresponding adjacent normal tissues were obtained from patients with PC who underwent surgery between January 2016 and December 2016 at the First Affiliated Hospital of Nanchang University (Nanchang, China). None of the patients received preoperative radiotherapy or chemotherapy. This study was approved by the Institutional Review Board at First Affiliated Hospital of Nanchang University. Written informed consent was obtained from each subject prior to recruitment in accordance with the Declaration of Helsinki.

### Bioinformatic Analysis

CircRNA expression profiles related to PC GSE69362 were obtained from the Gene Expression Omnibus (GEO) database (https://www.ncbi.nlm.nih.gov/gds/). Differential analysis was performed using the GEO2R online tool provided by NCBI. The threshold was set at | log_2_-fold change (FC) | > 0.2 and adjusted *P*-value < 0.05.

### Cell Culture

Human PC cell lines (PANC-1, SW1990, Mia PaCa-2, and BxPC-3), pancreatic duct epithelial cell line (HPDE6-C7), and mouse PC cell line Panc02 were purchased from the Type Culture Collection of the Chinese Academy of Sciences. Human PC cell line Capan-2 was purchased from the ATCC. The cells were maintained in DMEM supplemented with 10% FBS and penicillin-streptomycin (Gibco, Grand Island, NY) at 37°C under a 5% CO_2_ atmosphere.

### Quantitative Real-time PCR

Total RNA was isolated from snap-frozen fresh samples and cell lines using TRIzol Reagent (Invitrogen, CA, USA) and reverse transcribed using a PrimeScript RT Reagent Kit (Invitrogen, CA, USA). Quantitative PCR was performed using SYBR Premix Ex Taq (Takara, Dalian, China) in accordance with the manufacturer’s instructions. The expression U6 was used as an internal miRNA control and glyceraldehyde-3-phosphate dehydrogenase (GAPDH) was used as an internal mRNA control. The relative gene expression was determined using the 2^-ΔΔCt^ method. The primer sequences are listed in **Supplementary Table 1**.

### Western Blotting

The cells were lysed using RIPA buffer (Beyotime, Guangzhou, China) containing protease inhibitors. The protein concentrations were detected using a BCA Protein Assay Kit (Pierce, Rockford, IL, USA). Protein samples were separated by 10% SDS-PAGE, transferred to PVDF membranes, and then incubated with primary antibodies at 4°C overnight and secondary antibodies for 2 hours at room temperature. Finally, the proteins were visualized with enhanced chemiluminescence reagents (Millipore, USA). The primary antibodies against PD-L1 (ab233482, 1:1000) and GAPDH (ab8245, 1:2000) antibodies were obtained from Abcam (Cambridge, MA, USA). The relative protein levels were analyzed using Image J software.

### Lentivectors and Plasmid Transfection

Lentiviruses encoding oe-circRNA46523 (overexpression of hsa_circ_0046523), sh-circRNA46523 (down-expression of hsa_circ_0046523) and their respective negative control were synthesized by GenePharma (Shanghai, China) and then infected Mia PaCa-2, and BxPC-3 cells. The infection efficiency was confirmed by qRT-PCR. To generate stably transfected cells, the cells were treated with 2 μg/mL of puromycin (Sigma, St. Louis) for two weeks, then GFP-positive cells were selected for subsequent assays. hsa-miR-148a-3p mimic and inhibitor and their respective negative control vector were purchased from GenePharma and then transfected using Lipofectamine 3000 (Invitrogen). The transfection efficiency was confirmed by qRT-PCR.

### Cell Proliferation, Invasion and Migration Assay

Cell proliferation was measured using the CCK-8 assay. Stably transfected Mia PaCa-2 and BxPC-3 cells were seeded in 96-well plates at 5,000 cells/well. At the indicated time points (0 h, 24 h, 48 h, 72 h, and 96 h), 10 μL CCK-8 reagent (KeyGEN Biotech Co., Ltd., Nanjing, China) was added to each well and incubated for 1 h at 37°C away from light. The optical density (OD) values were determined using a microplate reader at 450 nm. Each assay was performed in triplicate.

Cell migration and invasion capacity were detected by Transwell assay. Transwell filters coated with Matrigel (BD Biosciences, USA) were used for the tumor cell invasion assay, while Transwell filters without Matrigel were used for the migration assay. Briefly, 5×10^5^ cells were added to the upper chambers with FBS-free DMEM. And DMEM containing 15% FBS were added into lower chambers. After incubation at 37°C for 24 h, cells in the upper chamber were gently removed with a cotton swab. The migrated or invaded cells were fixed with 4% polyformaldehyde and visualized by staining with 0.1% crystal violet. The number of migrated or invaded cells in five randomly selected fields was counted under a microscope (Olympus, Tokyo, Japan).

### Co-Cultured System and Flow Cytometry Assay

Peripheral blood mononuclear cells (PBMCs) were isolated from healthy donors and prepared following the manufacturer’s instructions. Briefly, PBMCs were isolated from 15 mL of venous blood using Ficoll-Hypaque. After centrifugation, PBMCs were collected from the interface and washed with RPMI 1640 medium. Then, PBMCs were cultured in with RPMI 1640 supplemented with 10% FBS and 10 units/mL IL-2 (PE Protech).

PC cells were cultured to 60%-70% confluence and co-cultured with PBMCs. PBMCs were cultured in RPMI1640 medium containing 10% FBS, supplemented with 300U/mL IL-2, human CD3 monoclonal antibody (10 μg/mL, 200 μL/well), and human CD28 monoclonal antibody (10 μg/mL, 200 μL/well) for 72 h. Activated PBMCs were seeded into the upper chamber of the Transwell at 1×10^6^ cells/well, and the Transwell chamber and PBMCs were transferred into a 6-well plate with PC cells at a concentration of 2×10^5^ cells/well. The cells were further co-cultured for an additional 72 h. In the specified group, cells were exposed to 20 ug/uL PD-L1 neutralizing antibody (R&D Systems). The percentage of CD4^+^ T cells (CD3^+^ and CD4^+^), CD8^+^ T cells (CD3^+^ and CD8^+^), and Tregs (CD4^+^, CD25^+^, and FOXP3^+^) among the total PBMCs, and the PD-L1 (PD-L1^+^) membrane expression of PC cells were analyzed with a FACScan apparatus (Becton Dickinson). Further, the CD8^+^ T cell apoptosis (annexin V^+^ and 7-AAD^+^), CD8^+^ T cell exhaustion (PD-1^+^ and Tim3^+^), and CD8^+^ T cell function (IFNγ^+^) were analyzed. The data were analyzed using FCS Express 6. Related flow cytometry antibodies are listed in **Supplementary Table 2**.

### Enzyme-Linked Immunosorbent Assay

The supernatant of the PBMCs co-cultured in the upper chamber was harvested after centrifugation at 700 g for 5 min at 4°C. IFN-γ, IL-2, IL-10, and TGF-β levels in the supernatant were measured using enzyme-linked immunosorbent assay (ELISA) commercial kits (Rapidbio) according to manufacturer’s instructions. The measurements were repeated with different dilutions to confirm the validity of the analyses.

### Dual-Luciferase Reporter Assay

The hsa_circ_0046523 binding sites to miR-148a-3p were predicted by the online software, StarBase3.0 (http://starbase.sysu.edu.cn/). PD-L1 was revealed to be a potential target of miR-148a-3p using TargetScan 7.2 (http://www.targetscan.org/vert_72/). The full-length sequence of hsa_circ_0046523 or the 3’UTR of PD-L1 containing the predicted binding sites of miR-148a-3p were amplified using PCR, and cloned into the XhoI and XbaI sites downstream of the Firefly luciferase gene in the pmirGLO vector (Promega, Madison) to generate the luciferase reporter vectors, hsa_circ_0046523-wt and PD-L1-3’UTR-wt, respectively. To test the binding specificity, the corresponding mutant type hsa_circ_0046523 or 3’UTR of PD-L1 lacking the miR-148a-3p binding sites were also cloned into the pmirGLO vector to form the reporter vectors, and named hsa_circ_0046523-mut and PD-L1-3’UTR-mut, respectively. BxPC-3 or Mia PaCa-2 cells were seeded into 24-well plates and co-transfected with 50 nM miR-148a-3p mimic or its negative control using Lipofectamine 3000, together with 100 ng of the indicated luciferase reporter vector. After 48 h, the luciferase activity in the transfected cells was detected using a Dual Luciferase^®^ Reporter Assay kit (Promega, Madison, USA) in accordance with the manufacturer’s instructions. Firefly luciferase activity was measured and normalized based on Renilla luciferase activity.

### *In Vivo* Experiments

C57BL/6 mouse and its synergic PC cell line Panc02 were used to established an immunocompetent murine PC model. Briefly, 4–6-week-old C57BL/6 male mice were purchased from the Animal Center of Nanjing University (Nanjing, China). Lentiviruses (GV229) encoding mmu-miR-148a-3p and its respective negative control were synthesized by GenePharma (Shanghai, China) and then infected Panc02 cells. Panc02 cells stably expressed mmu-miR-148a-3p, or corresponding controls were subcutaneously injected into the mice (1 × 10^7^ cells per mouse), with six mice each group. Tumor volumes were measured every 3 days for 30 days from the first injection using the formula: width^2^ × length/2. Mice were sacrificed at the last measurement, the tumors were weighed, and subsequent assays were performed. The expression of miR-148a-3p and PD-L1 mRNA in tumor tissues were detected by qRT-PCR, and the level of PD-L1 protein expression was detected by Western blotting. Immunohistochemistry (IHC) was used to detect the expression of PD-L1 (Abcam; ab233482, 1:100) and Ki67 (Abcam; ab279653, 1:100) in the tumor tissues. The overall staining scores were computed by multiplying the scores for staining intensity and extent of staining. The staining intensity was scored as 0 (no staining), 1 (weak staining), 2 (moderate staining), or 3 (strong staining). And the extent of staining was scored as 1 (<10% staining), 2 (10–40% staining), or 3 (>40% staining). The percentage of CD4^+^ T cells, CD8^+^ T cells, and Tregs in tumor tissues, as well as the apoptotic rate of CD8^+^ T cells, the level of IFN-γ secreted by CD8^+^ T cells, and the level of CD8^+^ T cell exhaustion were analyzed by FACS. All animal experimental procedures were performed in compliance with the institutional ethical requirements and were approved by the First Affiliated Hospital of Nanchang University Committee for the Use and Care of Animals.

### Statistical Analysis

All data were statistically analyzed and graphed using SPSS 24.0 (IBM, Corp., Armonk, NY) and GraphPad 8.0 software (GraphPad, San Diego, CA). All values were expressed as the mean ± SD. Differences between two groups were analyzed using t-tests or Student’s t-tests. Multiple group comparisons were performed using one-way analysis of variance (ANOVA) followed by Student-Newman-Keuls (S-N-K) method as the *post hoc* test. The enumeration data was expressed by n (%), and a χ^2^ test or Fisher’s exact test were used to calculate statistical differences. A Kaplan-Meier curve was used to evaluate the relationship between the expression of hsa_circ_0046523 and the overall survival of PC patients followed by Log-Rank test. Pearson’s correlation test was used for correlation analyses. A threshold of *P* < 0.05 was considered to be statistically significant.

## Results

### Differential CircRNA Expression Profile in PC

**Figure 1:**
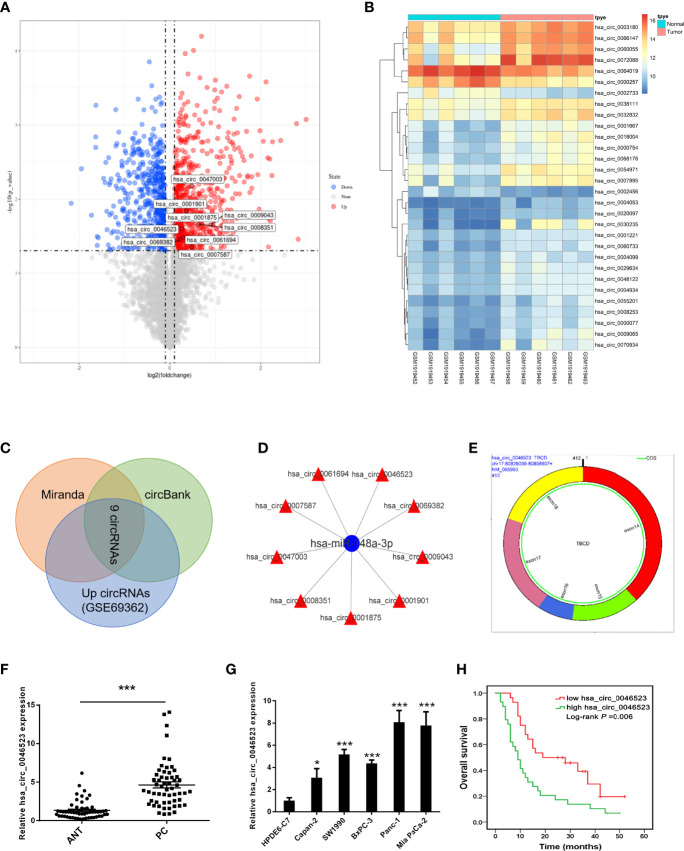
CircRNA profiling and the expression of hsa_circ_0046523 in PC. **(A)** Volcano map of differentially expressed circRNAs in PC tissues. **(B)** Cluster analysis of the expression levels of the top 30 upregulated circRNAs in PC tissues. **(C)** Predicted circRNAs with binding sites to miR-148a-3p from Miranda software and circBank database. **(D)** Network construction diagram of miR-148a-3p and circRNAs. **(E)** Basic characteristics of hsa_circ_0046523. **(F)** Relative hsa_circ_0046523 expression in 57 pairs of PC and ANT specimens. **(G)** Relative hsa_circ_0046523 expression in five PC cell lines (Capan-2, SW1990, BxPC-3, Panc-1 and Mia Paca-2) and the normal HPDE6-C7 cell line. **(H)** Kaplan–Meier analysis of the correlation between hsa_circ_0046523 expression and overall survival of PC patients. Data were expressed as means ± SD of three independent experiments. *P < 0.05, ***P < 0.001.

A total of 914 circRNAs were differentially expressed in PC tissues based on GSE69362 dataset, including 492 (53.8%) up-regulated, 422 (46.2%) down-regulated circRNAs. The volcano map showed all differentially expressed circRNAs in the PC tissues (**Figure 1A**) and the heat map listed the top 30 up-regulated circRNAs (**Figure 1B**). Subsequently, we searched for circRNAs with miR-148a-3p binding sites and highly expressed in PC through Miranda software and circBank database predictions. A total of 9 circRNAs were screened out, including hsa_circ_0008351, hsa_circ_0047003, hsa_circ_0007587, hsa_circ_0061694, hsa_circ_0046523, hsa_circ_0069382, hsa_circ_0009043, hsa_circ_0001901, and hsa_circ_0001875 (**Figures 1C, D**). Finally, we selected hsa_circ_0046523 to verify it expression in PC tissues and cell lines. Based on circBase, hsa_circ_0046523 is located at chr17: 80828099-80858607, containing 412 nucleotides. It is an exon-derived circRNA and its gene symbol is TBCD (**Figure 1E**).

### Hsa_circ_0046523 Is Overexpression in PC and Associated With Poor Prognosis

Using 57 pairs of patient samples from our institution, we further verified the overexpression of hsa_circ_0046523 in PC tissues by qRT-PCR (**Figure 1F**). Consistently, hsa_circ_0046523 was markedly upregulated in five PC cell lines (Panc-1, SW1990, Mia Paca-2, BxPC-3 and Canpan-2) relative to normal pancreatic duct epithelial cell line HPDE6-C7 (**Figure 1G**). In addition, hsa_circ_0046523 expression was revealed to be significantly higher in patients with advanced tumor differentiation, T stage, TNM stage and lymph node metastasis, after the 57 cases were divided into low (n=28) and high expression (n=29) according to the median expression level of hsa_circ_0046523 (**Table 1**). Moreover, we found that high expression of hsa_circ_0046523 was correlated with poorer overall survival (**Figure 1H**). All evidence suggests that hsa_circ_0046523 could be a potential functional circRNA in the progression of PC.

**Table 1:** Correlation between expression of hsa_circ_0046523 and clinicopathological parameters of 57 PC patients.

Variables	n	hsa_circ_0046523		χ^2^	*P*-value
		High expression	Low expression		
Sex				0.030	0.862
Male	21	11 (52.4%)	10 (47.6%)		
Female	36	18 (50.0%)	18 (50.0%)		
Age (years)				0.427	0.514
≥60	31	17 (54.8%)	14 (45.2%)		
<60	26	12 (46.2%)	14 (53.8%)		
T staging				8.278	0.016*
T1	20	5 (25.0%)	15 (75.0%)		
T2	25	16 (64.0%)	9 (36.0%)		
T3	12	8 (66.7%)	4 (33.3%)		
Nerve infiltration				1.707	0.191
Yes	43	24 (55.8%)	19 (44.2%)		
No	14	5 (35.7%)	9 (64.3%)		
Histological grade				10.703	0.001*
High	17	3 (17.6%)	14 (82.4%)		
Low / Medium	40	26 (65.0%)	14 (35.0%)		
Lymph node status				6.440	0.011*
Negative	14	3 (21.4%)	11 (78.6%)		
Positive	43	26 (60.5%)	17 (39.5%)		
TNM staging				6.467	0.039*
I	10	2 (20.0%)	8 (80.0%)		
II	38	20 (52.6%)	18 (47.4%)		
III	9	7 (77.8%)	2 (22.2%)		

*P < 0.05.

### Overexpression of Hsa_circ_0046523 Promoted the Proliferation, Migration and Invasion of PC Cells

**Figure 2:**
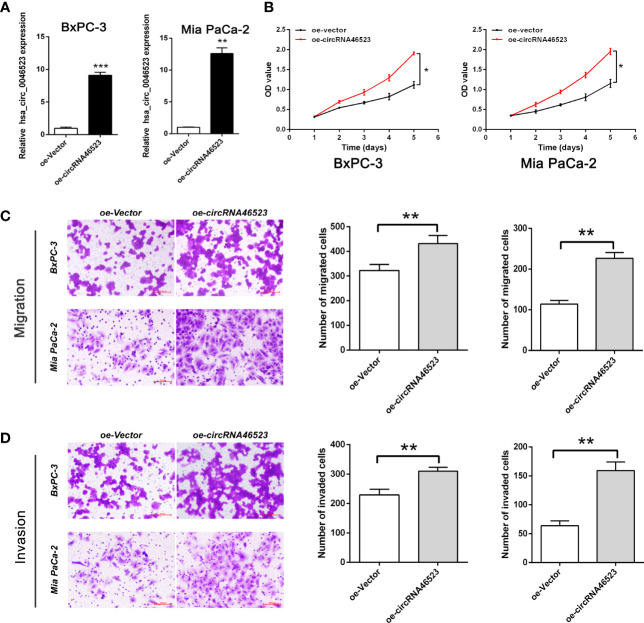
Hsa_circ_0046523 overexpression promoted the proliferation, migration and invasion of PC cells. **(A)** After infecting lentivirus encoding oe-circRNA46523, the expression level of hsa_circ_0046523 in BxPC-3 and Mia PaCa-2 cells was confirmed using RT-qPCR analysis. **(B)** CCK‐8 assays were conducted to estimate the effects of hsa_circ_0046523 overexpression on the proliferation of BxPC-3 and Mia PaCa-2 cells. **(C, D)** Transwell assays were conducted to estimate the effects of hsa_circ_0046523 overexpression on the cell migration and invasion capacities of BxPC-3 and Mia PaCa-2 cells. Data were expressed as means ± SD of three independent experiments. *P < 0.05, **P < 0.01, ***P < 0.001.

We performed a CCK-8 assay and Transwell assay to measure the effect of hsa_circ_0046523 on the proliferation, migration and invasion capacity of PC cells (BxPC-3 and Mia PaCa-2). The efficacy of oe-circRNA4652 and shRNA-circRNA46523 infection were confirmed by qRT-PCR (**Figure 2A** and **Supplementary Figure 1A**). CCK-8 assay indicated that forced expression of hsa_circ_0046523 significantly promoted cell proliferation (**Figure 2B**), while hsa_circ_0046523 silencing significantly inhibited cell proliferation (**Supplementary Figure 1B**). Transwell assay demonstrated that hsa_circ_0046523 overexpression promoted the migration and invasion capacities (**Figures 2C, D**), while hsa_circ_0046523 knockdown significantly impaired the migration and invasion capacities of PC cells (**Supplementary Figures 1C, D**). These findings suggested that hsa_circ_0046523 overexpression could promote the malignant behaviors of PC cells *in vitro*.

### Overexpression of Hsa_circ_0046523 Mediated an Immunosuppressive Microenvironment in Co-Cultured System

**Figure 3:**
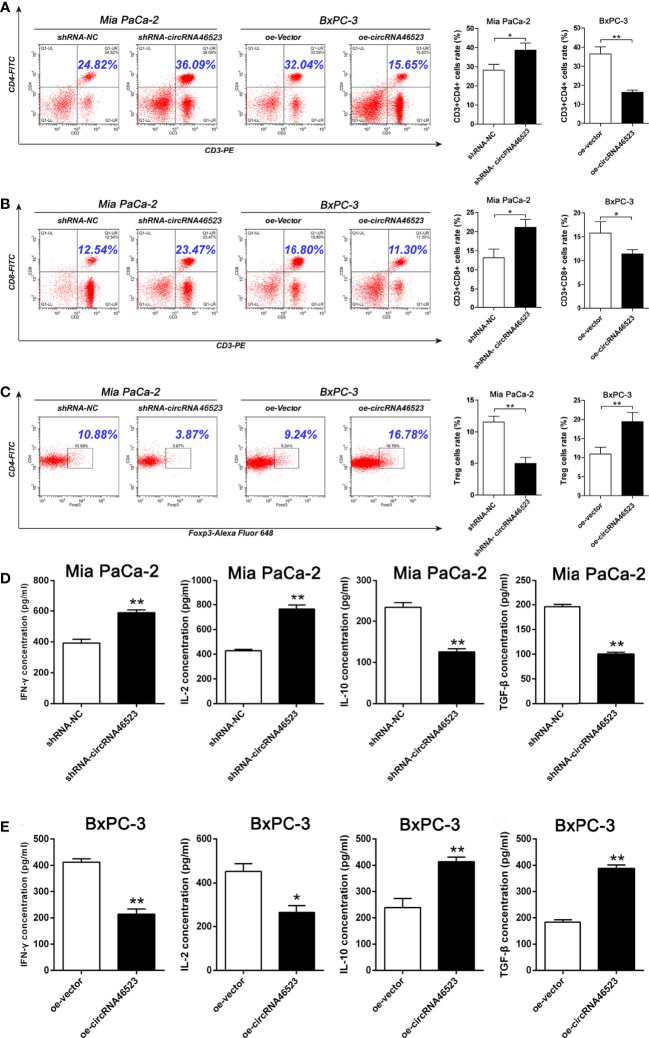
Hsa_circ_0046523 mediated an immunosuppressive microenvironment of PC in the co-cultured system. PC cells overexpressing or knocking down hsa_circ_0046523 were co-cultured with PBMCs, **(A–C)** Flow cytometry analysis of the proportion of CD4^+^, CD8^+^ T cells and Tregs in PBMCs. **(D, E)** ELISA analysis of the levels of the immune effect cytokines IFN-γ and IL-2, and immunosuppressive cytokines IL-10 and TGF-β in the supernatant of PBMC cells. Data were expressed as means ± SD of three independent experiments. *P < 0.05, **P < 0.01.

To investigate the effect of hsa_circ_0046523 on the immune microenvironment of PC, PC cells (BxPC-3 and Mia PaCa-2) stably expressed oe-circRNA46523, sh-circRNA46523 or their corresponding controls were co-cultured with PBMCs to simulate the status of the PC immune microenvironment *in vitro*. Flow cytometry results showed that hsa_circ_0046523 knockdown markedly increased the proportion of CD4^+^ and CD8^+^ T cells (**Figures 3A–B**), and decreased the proportion of Tregs in PBMCs co-cultured with PC cells (**Figure 3C**); While hsa_circ_0046523 overexpression showed an opposite effect (**Figures 3A–C**). Meanwhile, hsa_circ_0046523 knockdown markedly inhibited the apoptosis and exhaustion of CD8^+^ T cells, whereas hsa_circ_0046523 overexpression promoted the apoptosis and exhaustion of CD8^+^ T cells in PBMCs co-cultured with PC cells (**Supplementary Figures 2A–F**). In addition, hsa_circ_0046523 knockdown improved the function of CD8^+^ T cells, while hsa_circ_0046523 overexpression suppressed the function of CD8^+^ T cells in PBMCs co-cultured with PC cells (**Supplementary Figures 2G–I**). Results of ELISA assay showed that hsa_circ_0046523 knockdown markedly increased the expression levels of immune effector cytokines IFN-γ and IL-2, and decreased the expression levels of immunosuppressive cytokines IL-10 and TGF-β (**Figure 3D**). However, hsa_circ_0046523 overexpression exerted an opposite effect (**Figure 3E**). Collectively, these results suggested that hsa_circ_0046523 could mediated an immunosuppressive microenvironment through regulating the proportion of T cells, function of CD8^+^ T cells, and secretion of immunomodulatory cytokines.

### Hsa_circ_0046523 Functions as the Sponge of MiR-148a-3p

**Figure 4:**
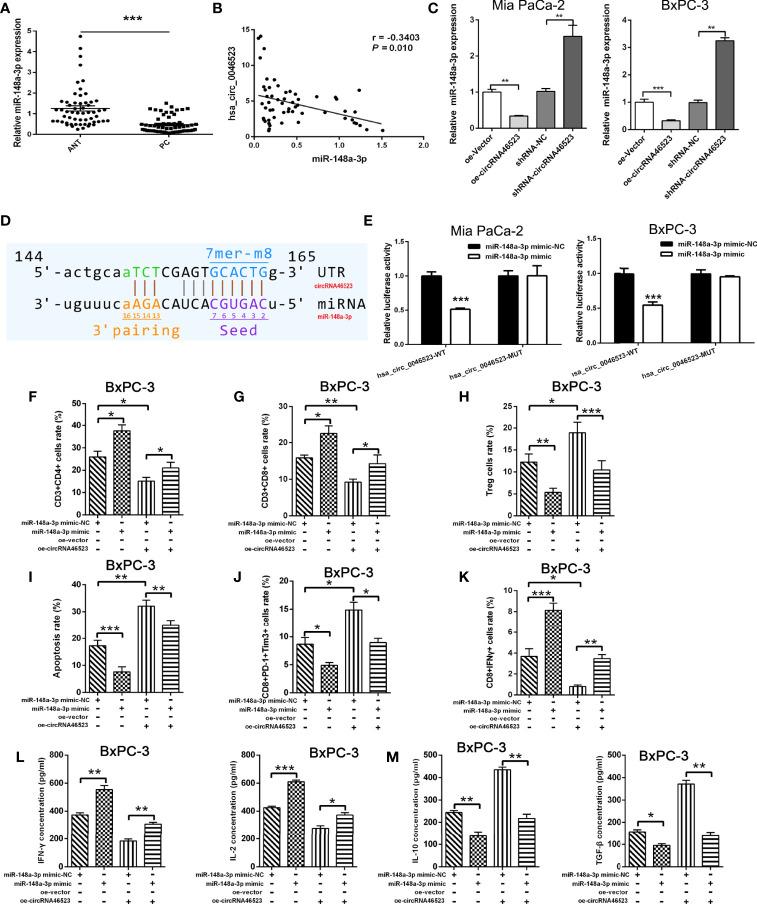
Hsa_circ_0046523 functions as a sponge of miR-148a-3p. **(A)** qRT-PCR analysis of the expression of miR-148a-3p in 57 pairs of PC and ANT specimens. **(B)** The expression level of miR-148a-3p is inversely correlated with hsa_circ_0046523 in PC tissues (Pearman’s correlation analysis, r =-0.3789, *P* =0.004). **(C)** qRT-PCR analysis of miR-148a-3p expression after hsa_circ_0046523 overexpression or knockdown. **(D)** The targeted binding site of hsa_circ_0046523 and miR-148a-3p. **(E)** Luciferase activities of hsa_circ_0046523-wt and hsa_circ_0046523-mut constructs when transfecting with miR-148a-3p mimic in PC cells. **(F–H)** PC cells with upregulated miR-148a-3p or combined with overexpressed hsa_circ_0046523 were co-cultured with PBMC cells. Flow cytometry analysis of the proportion of CD4^+^, CD8^+^ T cells and Tregs in PBMCs after co-culture. **(I–K)** PC cells with upregulated miR-148a-3p or combined with overexpressed hsa_circ_0046523 were co-cultured with PBMC cells. Flow cytometry analysis of apoptosis, exhaustion and function of CD8^+^ T cells in PBMCs after co-culture. **(L, M)** PC cells with upregulated miR-148a-3p or combined with overexpressed hsa_circ_0046523 were co-cultured with PBMC cells. ELISA analysis of the levels of the immune effect cytokines IFN-γ and IL-2, and immunosuppressive cytokines IL-10 and TGF-β in the supernatant of PBMC cells after co-culture. Data were expressed as means ± SD of three independent experiments. *P < 0.05, **P < 0.01, ***P < 0.001.

To validate the interaction between hsa_circ_0046523 and miR-148a-3p, the expression level of miR-148a-3p in 57 paired PC tissues and adjacent normal tissues was detected by qRT-PCR. The results showed that miR-148a-3p was significantly downregulated in PC tissues and was inversely correlated with hsa_circ_0046523 (**Figures 4A, B**). Meanwhile, hsa_circ_0046523 overexpression markedly downregulated the expression of miR-148a-3p, and hsa_circ_0046523 silencing upregulated the expression of miR-148a-3p in Mia PaCa-2 and BxPC-3 cells (**Figure 4C**). Further luciferase reporter assays showed that miR-148a-3p could directly bind to hsa_circ_0046523 (**Figures 4D, E**). Moreover, miR-148a-3p overexpression upregulated the proportion of CD4^+^ and CD8^+^ T cells and downregulated the proportion of Tregs in PBMCs co-cultured with PC cells (**Figures 4F–H**), while miR-148a-3p depletion executed an opposite effect (**Supplementary Figures 3A–C**). Meanwhile, miR-148a-3p overexpression impaired the apoptosis and exhaustion of CD8^+^ T cells (**Figures 4I, J**), improved the function of CD8^+^ T cells (**Figure 4K**), upregulated the expression of immune effector cytokines IFN-γ and IL-2 (**Figure 4L**), and downregulated the expression of immunosuppressive cytokines IL-10 and TGF-β (**Figure 4M**). While miR-148a-3p depletion caused an opposite effect (**Supplementary Figures 3D–H**). Rescue experiments demonstrated that the decreased proportion of CD4^+^ and CD8^+^ T cells, function of CD8^+^ T cells, and expression of immune effector cytokines, and the increased proportion of Tregs, apoptosis and exhaustion of CD8^+^ T cells, and expression of immunosuppressive cytokines caused by hsa_circ_0046523 overexpression, were effectively restored by miR-148a-3p upregulation (**Figures 4F–M**). These findings indicated that miR-148a-3p upregulation could partially reverse the anti-immune function of hsa_circ_0046523 overexpression in PC. Likewise, the rescue experiments demonstrated that miR-148a-3p downregulation could partially reverse the immune-promoting function of hsa_circ_0046523 knockdown in PC (**Supplementary Figures 3A–H**). Taken together, these results confirmed that hsa_circ_0046523 mediated the formation of the PC immunosuppressive microenvironment by sponging miR-148a-3p.

### Hsa_circ_0046523/MiR-148a-3p/PD-L1 Regulation Axis in PC

**Figure 5:**
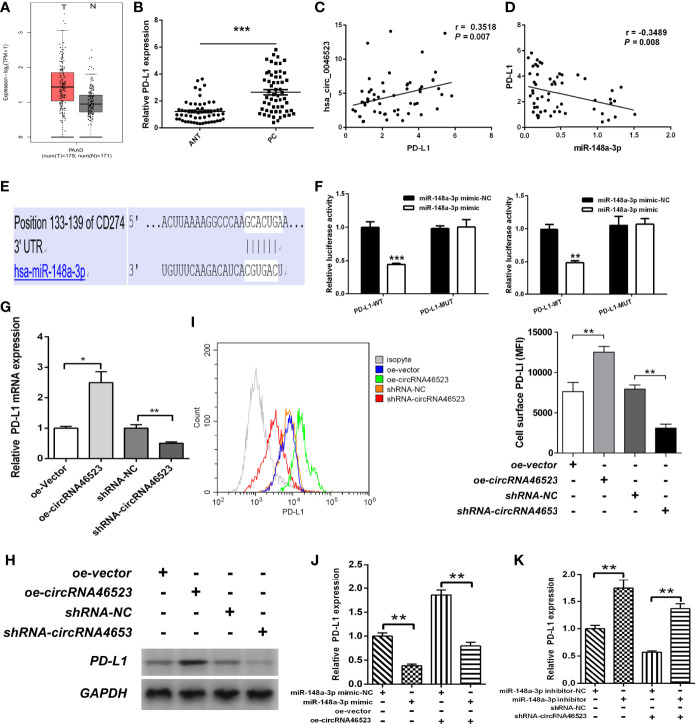
Hsa_circ_0046523 regulated PD-L1 expression by sponging miR-148a-3p. **(A)** GEPIA database was used to analyze the expression of PD-L1 in PC tissues. **(B)** qRT-PCR analysis of the expression of PD-L1 in 57 pairs of PC and ANT specimens. **(C, D)** The expression level of PD-L1 is positively correlated with hsa_circ_0046523 (Pearson’s correlation analysis, r = 0.3111, *P* = 0.019), while negatively correlated with miR-148a-3p in PC tissues (Pearson’s correlation analysis, r = -0.3516, *P* = 0.007). **(E)** The targeted binding site of PD-L1 and miR-148a-3p. **(F)** Luciferase activities of PD-L1-3’UTR-wt and PD-L1-3’UTR-mut constructs when transfecting with miR-148a-3p mimic in PC cells. **(G, H)** qRT-PCR and WB analysis of PD-L1 expression in PC cells after hsa_circ_0046523 overexpression or knockdown. **(I)** Flow cytometry analysis of the membrane expression of PD-L1 in PC cells after hsa_circ_0046523 overexpression or knockdown. **(J)** qRT-PCR analysis the expression of PD-L1 in PC cells after upregulation of miR-148a-3p or combined with hsa_circ_0046523 overexpression. **(K)** qRT-PCR analysis the expression of PD-L1 in PC cells after down-regulation of miR-148a-3p or combined with hsa_circ_0046523 knockdown. Data were expressed as means ± SD of three independent experiments. *P < 0.05, **P < 0.01, ***P < 0.001.

Using the GEPIA database, we found that PD-L1 was highly expressed in PC tissues (**Figure 5A**). Based on our samples, PD-L1 expression was significantly upregulated in PC tissues, and was positively correlated with hsa_circ_0046523 expression, and was negatively correlated with miR-148a-3p expression (**Figures 5B–D**). Dual luciferase reporter assay showed that miR-148a-3p could directly bind to the 3’UTR of PD-L1 (**Figures 5E, F**). In addition, qRT-PCR and Western blotting analysis indicated that overexpression of hsa_circ_0046523 markedly increased the expression of PD-L1, while knockdown of hsa_circ_0046523 decreased the expression of PD-L1 in Mia PaCa-2 and BxPC-3 cells (**Figures 5G, H**). Flow cytometry results showed overexpression of hsa_circ_0046523 upregulated PD-L1 membrane expression, whereas knockdown of hsa_circ_0046523 downregulated PD-L1 membrane expression in PC cells (**Figure 5I**). miR-148a-3p mimic transfection significantly inhibited PD-L1 expression, and reversed the increased PD-L1 expression caused by hsa_circ_0046523 overexpression in PC cells (**Figure 5J**). However, miR-148a-3p inhibitor transfection promoted PD-L1 expression, and restored the decreased PD-L1 expression caused by hsa_circ_0046523 knockdown in PC cells (**Figure 5K**). These results indicated that there existed a hsa_circ_0046523/miR-148a-3p/PD-L1 regulation axis in PC.

**Figure 6:**
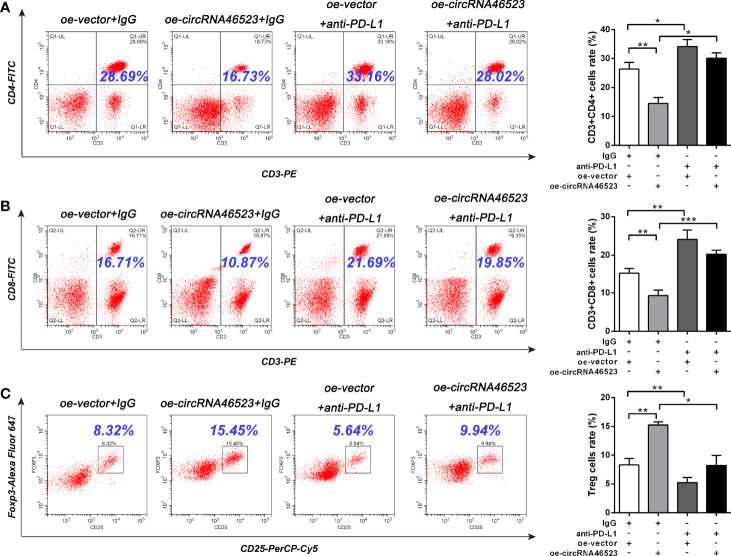
Hsa_circ_0046523 regulated the proportion of CD4^+^ T cells, CD8^+^ T cells and Tregs in PBMCs *via* PD-L1. PC cells with hsa_circ_0046523 overexpression were co-cultured with PBMCs, and PD-L1 neutralizing antibody (anti-PD-L1) was added to the co-culture system. **(A–C)** Flow cytometry analysis of the proportion of CD4^+^, CD8^+^ T cells and Tregs in PBMCs after co-culture. Data were expressed as means ± SD of three independent experiments. *P < 0.05, **P < 0.01, ***P < 0.001.

**Figure 7:**
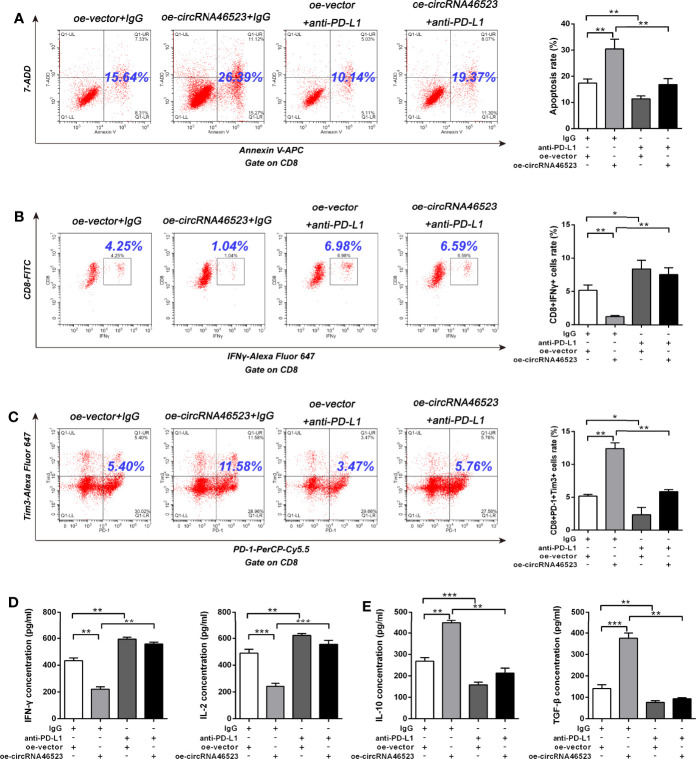
Hsa_circ_0046523 regulated the apoptosis, function and exhaustion of CD8^+^ T cells, and the expression of immunoregulatory cytokines *via* PD-L1. PC cells with hsa_circ_0046523 overexpression were co-cultured with PBMCs, and PD-L1 neutralizing antibody (anti-PD-L1) was added to the co-culture system. **(A–C)** Flow cytometry analysis of apoptosis, function and exhaustion of CD8^+^ T cells in PBMCs after co-culture. **(D, E)** ELISA analysis of the levels of the immune effect cytokines IFN-γ and IL-2, and immunosuppressive cytokines IL-10 and TGF-β in the supernatant of PBMC cells after co-culture. Data were expressed as means ± SD of three independent experiments. *P < 0.05, **P < 0.01, ***P < 0.001.

To further confirm whether hsa_circ_0046523/miR-148a-3p inducing the immunosuppressive microenvironment was mediated by PD-L1, we designed an *in vitro* blocking experiment. The PD-L1 neutralizing antibody (anti-PD-L1) was added to the co-cultured system to block the mediating effect of PD-L1. Flow cytometry results showed that the decreased proportion of CD4^+^ and CD8^+^ T cells, and the increased proportion of Tregs caused by hsa_circ_0046523 overexpression, were effectively reversed by PD-L1 blocking (**Figures 6A–C**). Meanwhile, the increased apoptosis and exhaustion of CD8^+^ T cells, and the impaired function of CD8^+^ T cells caused by hsa_circ_0046523 overexpression, were also effectively reversed by PD-L1 blocking (**Figures 7A–C**). Moreover, the decreased expression of immune effector cytokines IFN-γ and IL-2, and the increased expression of immunosuppressive cytokines IL-10 and TGF-β caused by hsa_circ_0046523 overexpression, were effectively reversed by PD-L1 blocking (**Figures 7D, E**). These findings suggested that blocking PD-L1 could effectively counteract the anti-immune effects of hsa_circ_0046523 overexpression. Likewise, blocking PD-L1 effectively reversed the anti-immune function of miR-148a-3p downregulation in PC (**Supplementary Figures 4A–H**). Taken together, these results confirmed that the effect of hsa_circ_0046523 and miR-148a-3p on the PC immune microenvironment was dependent on PD-L1.

### MiR-148a-3p/PD-L1 Molecular Axis Functions *in Vivo*

**Figure 8:**
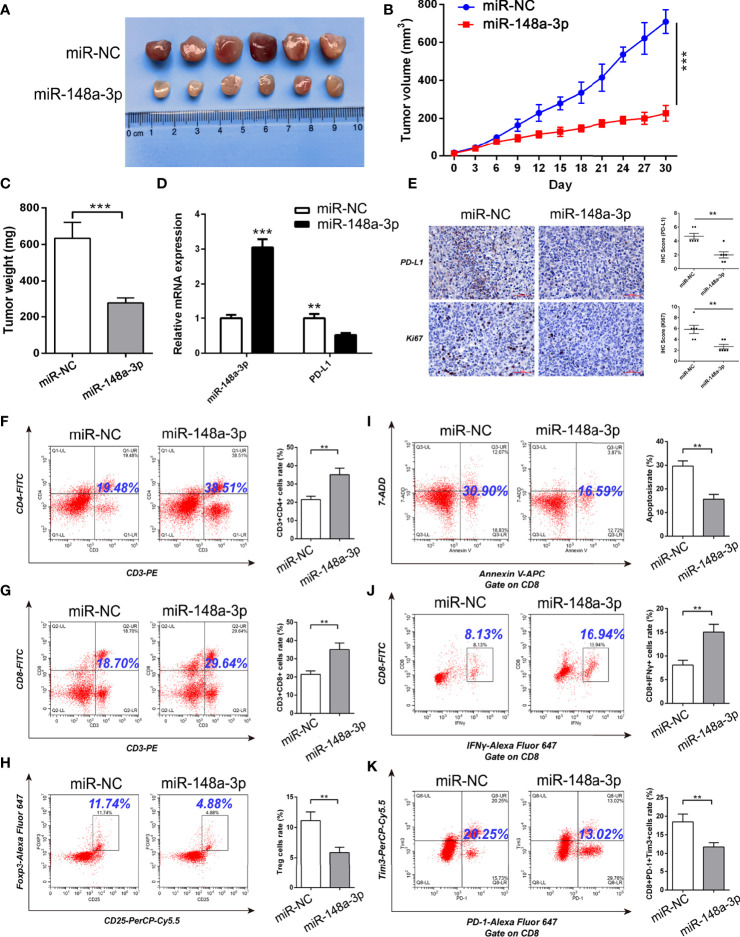
MiR-148a-3p inhibited the formation of PC immunosuppressive microenvironment *via* targeting PD-L1 in murine xenograft model. **(A)** Representative images of xenograft tumors. **(B, C)** Tumor growth curves and tumor weights. **(D)** qRT-PCR analysis of the expression of miR-148a-3p and PD-L1 in the tumor tissues. **(E)** IHC staining of PD-L1 and Ki67 in the tumor tissues. **(F–H)** Flow cytometry analysis of the proportion of CD4^+^, CD8^+^ T cells and Tregs in the tumor tissues. **(I–K)** Flow cytometry analysis of apoptosis, function and exhaustion of CD8^+^ T cells in the tumor tissues. Data were expressed as means ± SD of three independent experiments. **P < 0.01, ***P < 0.001.

Because the mouse-derived sequence of hsa_circ_0046523 remains unknown, it is difficult to artificially regulate the expression of hsa_circ_0046523 in murine xenograft model. Therefore, we focused on the function of miR-148a-3p/PD-L1 molecular axis *in vivo.* Based on an immunocompetent murine xenograft PC model, we observed that overexpression of miR-148a-3p significantly suppressed tumor growth (**Figures 8A–C**). qRT-PCR and IHC analysis showed that tumors with high expression of miR-148a-3p exhibited lower PD-L1 and Ki67 expression (**Figures 8D, E**). Meanwhile, FCM analysis showed that forced expression of miR-148a-3p significantly increased the proportion of CD4^+^ and CD8^+^ T cells, decreased the proportion of Tregs, reduced the apoptosis and exhaustion of CD8^+^ T cells, and enhanced the function of CD8^+^ T cells in xenograft tumors (**Figures 8F–K**). These results indicated that miR-148a-3p could inhibit the formation of PC immunosuppressive microenvironment *via* targeting PD-L1 *in vivo*.

## Discussion

CircRNAs represent a class of noncoding RNAs with a high degree of stability and participate in multiple biological processes in human cells (25). In recent years, accumulated evidence has suggested that circRNAs have oncogenic or tumor suppressor functions in a variety of human cancers, including PC (26–28). In 2016, Li et al. firstly investigated the expression profile of circRNAs in six PC tissues and paired adjacent normal specimens using microarray, and found that there were 351 differentially expressed circRNAs in PC tissues, including 209 up-regulation and 142 down-regulation circRNAs (23). Since then, more and more circRNAs were identified aberrant expression in PC and theirs’ functions also have been gradually recognized. These functions are involved in regulation of cellular processes in PC, including proliferation, apoptosis, cell cycle, migration, invasion, metastasis, epithelial to mesenchymal transition (EMT), angiogenesis, drug resistance and immune escape (28). For example, Wong et al. identified 169 differentially expressed circRNAs in PC cells by circRNA sequencing. Among these dysregulated circRNAs, CircFOXK2 was significantly upregulated in PC cells and 63% of primary tumors. Mechanistically, CircFOXK2 promoted cell proliferation, migration and invasion of PC *via* modulation of miR-942/ANK1/GDNF/PAX6 axis (29). The other study revealed that circBFAR expression was upregulated in PC tissues, and the ectopic expression of circBFAR was positively correlated with TNM stage and poorer prognosis of patients with PC (30). However, the function and mechanism of circRNAs in regulating immune microenvironment of PC remain largely unclear. At the present study, we firstly demonstrated that hsa_circ_0046523 expression was remarkably upregulated in PC tissues and cell lines. Moreover, high expression of hsa_circ_0046523 was correlated with advanced pathological stage and poorer prognosis, which suggested that hsa_circ_0046523 might be involved in the progression of PC. *In vitro*, forced expression of hsa_circ_0046523 promoted the proliferation, migration and invasion of PC cells. In addition, hsa_circ_0046523 overexpression could decrease the proportion of CD4^+^ and CD8^+^ T cells, increase the proportion of Tregs among PBMCs co-cultured with PC cells. Furthermore, hsa_circ_0046523 overexpression promoted the apoptosis and exhaustion of CD8^+^ T cells and the secretion of immunosuppressive cytokines, impaired CD8^+^ T cell function, and inhibited the secretion of effector cytokines. Mechanistically, hsa_circ_0046523 acted as a ceRNA sponging miR-148a-3p to regulate PD-L1 expression in PC.

Within the TIME of PC, the number and function of immune cells exist in a state of imbalance. That is, the immune effector cells like CD8^+^ T cells, CD4^+^ Th, DCs, and NK cells which have anti-tumor effects are reduced in number, whereas immunosuppressive cells such as Tregs, M2 type TAMs, and MDSCs are present in large numbers (4, 31). This situation makes tumor cells located in an immunosuppressive microenvironment, which promoting tumor cells escape from immune surveillance and resist immune attack. Unfortunately, the formation and regulatory mechanism of the PC immunosuppressive microenvironment are not fully elucidated, and its influence on tumor cells remains unclear till to now. At the present study, we used PBMCs and PC cells co-cultured system to simulate the TME of PC *in vitro*, in order to investigate whether hsa_circ_0046523 was involved in the formation of PC immunosuppressive microenvironment. Our data showed that hsa_circ_0046523 overexpression significantly decreased the proportion of CD4^+^ and CD8^+^ T cells and increased the proportion of Tregs in the PC immune microenvironment. At the same time, hsa_circ_0046523 promoted the apoptosis and exhaustion of CD8^+^ T cells, and impaired the function of CD8^+^ T cells. Moreover, hsa_circ_0046523 inhibited the secretion of immune effector cytokines IFN-γ and IL-2, and promoted the secretion of immunosuppressive cytokines IL-10 and TGF-β. Obviously, these evidences indicated that hsa_circ_0046523 take part in the forming process of PC immunosuppressive microenvironment through regulating the distribution and function of the immune cells, as well as immune cytokines.

**Figure 9:**
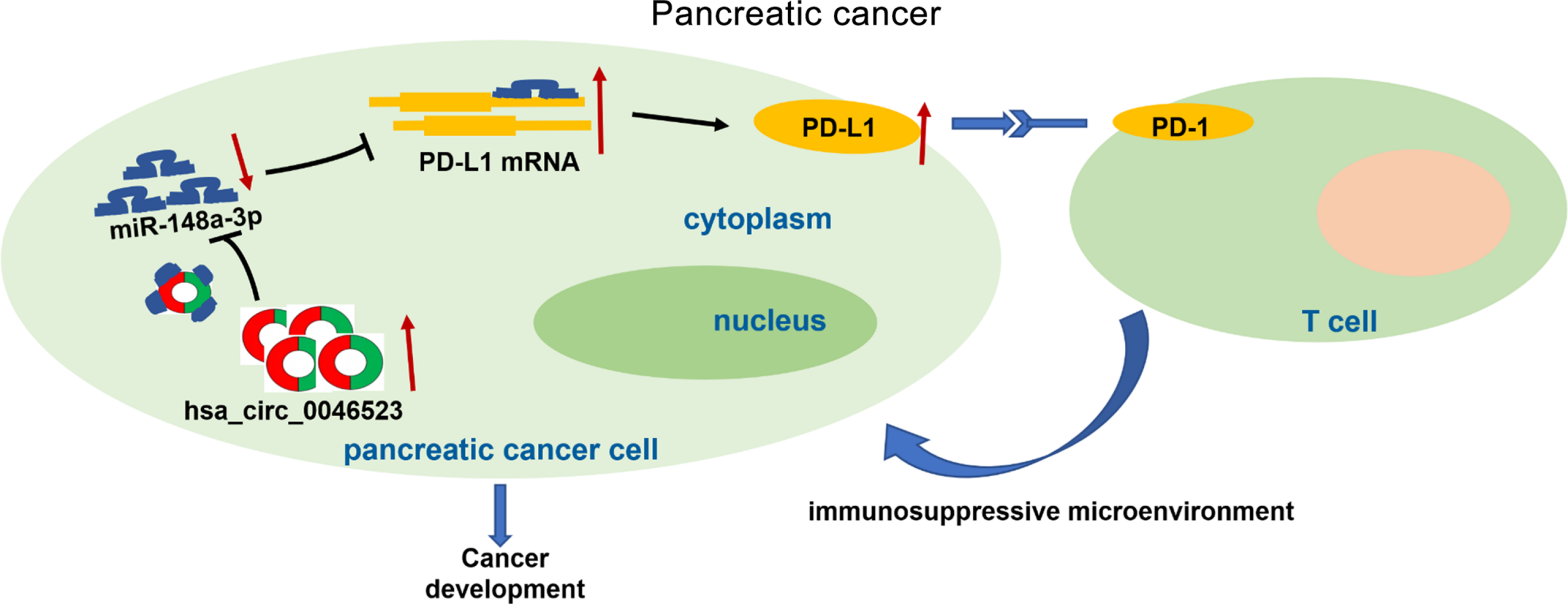
The schematic illustration of the potential molecular mechanism of hsa_circ_0046523 as a key regulator tumor immune microenvironment of PC. Hsa_circ_0046523 was remarkably upregulated in PC, and upregulated PD-L1 expression by sponging miR-148a-3p, which activating the PD-1/PD-L1 pathway, inducing the formation of immunosuppressive microenvironment of PC, and ultimately promoting the progression of PC.

As a co-stimulatory signal pathway of T cell immune response, PD-1/PD-L1 is an important target for tumor immunotherapy. Anti-PD-1/PD-L1 therapy has also shown favorable therapeutic effects in a variety of tumors. Recent studies have confirmed that PD-L1 was abnormally expressed in a variety of human tumor cells and was closely related to the clinical pathological parameters, as well as a poor prognosis (9, 32, 33). Previous studies have also shown that tumor cells and the tumor microenvironment could up-regulate PD-L1, activate PD-1/PD-L1 signaling pathways, inhibit T cell activation and proliferation, and induce T cell apoptosis (10, 11). Activation of the PD-1/PD-L1 signaling pathway could also maintain Tregs function and promote their development. Tumor cells expressing PD-L1 combined with locally infiltrated Tregs exerted a synergistic immunosuppressive effect, induced the formation of an immunosuppressive microenvironment, and promoted the immune escape of tumor cells (34–36). Our results revealed that PD-L1 was highly expressed in PC tissues, and the expression level of PD-L1 was negatively correlated with miR-148a-3p expression, and positively correlated with hsa_circ_0046523 expression in PC. The dual luciferase gene reporter experiment confirmed that miR-148a-3p could directly bind to hsa_circ_0046523 and 3’UTR of PD-L1 mRNA. These findings demonstrated that hsa_circ_0046523 functioned as a ceRNA for miR-148a-3p, in turn regulating the expression of PD-L1 in PC. Rescue experiments confirmed that miR-148a-3p upregulation could partially reverse the immunosuppressive function of hsa_circ_0046523 overexpression, and miR-148a-3p downregulation can partially reverse the immune-promoting function of hsa_circ_0046523 knockdown. *In vitro* blocking experiments showed that blocking PD-L1 could effectively counteract the immunosuppressive effects of hsa_circ_0046523 overexpression and miR-148a-3p downregulation. A subcutaneous xenograft tumor model in immunocompetent mice confirmed that miR-148a-3p overexpression inhibited PD-L1 expression, thereby regulating the proportion of CD4^+^ T cells, CD8^+^ T cells, and Tregs in the PC immune microenvironment, as well as the apoptosis, function, and exhaustion of CD8^+^ T cell. Collectively, these results suggested that hsa_circ_0046523 upregulated PD-L1 expression in PC through sponging miR-148a-3p, which induced the formation of an immunosuppressive microenvironment and promoted the immune escape of PC cells (**Figure 9**).

Undoubtedly, there are some limitations in this study. First of all, because the sequence of mice hsa_circ_0046523 remained unknown, it was difficult to verify the effect of hsa_circ_0046523 on PC immune microenvironment in mice xenograft model. Secondly, Panc02 is a chemically induced mouse-derived PC cell line, which cannot provide the most reliable TME status of human PC. Therefore, KPC or PC patient-derived xenograft (PDX) models should be applied for further validation.

In summary, this study firstly identified hsa_circ_0046523 as a tumor promotor and potential prognosis marker of PC. Furthermore, we confirmed that hsa_circ_0046523/miR-148a-3p/PD-L1 regulatory axis mediates PC immunosuppressive microenvironment and these molecules are expected to be new targets for remodeling tumor immune microenvironment of PC.

## Data Availability Statement

The original contributions presented in the study are included in the article/**Supplementary Material**. Further inquiries can be directed to the corresponding author.

## Ethics Statement

The studies involving human participants were reviewed and approved by the First Affiliated Hospital of Nanchang University Committee. The patients/participants provided their written informed consent to participate in this study.

## Author Contributions

WX and XF designed the experiments. XF, GS, ST, KF and YX carried out the experiments. YT, MZ and TX collected clinical specimens and followed up. XF, GS and WX analyzed the data. XF and WX wrote the manuscript. All authors approved the final manuscript.

## Funding

The study is supported by the National Natural Science Foundation of China (81860418), the Natural Science Foundation of Jiangxi Province (20202ACB206007), and the Key Research and Development Program of Jiangxi Province (20192BBG70035).

## Conflict of Interest

The authors declare that the research was conducted in the absence of any commercial or financial relationships that could be construed as a potential conflict of interest.

## Publisher’s Note

All claims expressed in this article are solely those of the authors and do not necessarily represent those of their affiliated organizations, or those of the publisher, the editors and the reviewers. Any product that may be evaluated in this article, or claim that may be made by its manufacturer, is not guaranteed or endorsed by the publisher.

## Supplementary Material

Supplementary Figure 1 Hsa_circ_0046523 knockdown inhibited the proliferation, migration and invasion of PC cells. **(A)** After infecting lentivirus encoding sh-circRNA46523, the expression level of hsa_circ_0046523 in BxPC-3 and Mia PaCa-2 cells was confirmed using RT-qPCR analysis. **(B)** CCK‐8 assays were conducted to estimate the effects of hsa_circ_0046523 knockdown on the proliferation of BxPC-3 and Mia PaCa-2 cells. **(C, D)** Transwell assays were conducted to estimate the effects of hsa_circ_0046523 knockdown on the cell migration and invasion capacities of BxPC-3 and Mia PaCa-2 cells. Data were expressed as means ± SD of three independent experiments. **P* < 0.05, ***P* < 0.01.

Supplementary Figure 2 Hsa_circ_0046523 regulated the apoptosis, exhaustion and function of CD8^+^ T cells in the co-cultured system. PC cells overexpressing or knocking down hsa_circ_0046523 were co-cultured with PBMC cells. **(A–C)** Flow cytometry analysis of the apoptosis of CD8^+^ T cells in PBMCs after co-culture. **(D–F)** Flow cytometry analysis of the exhaustion of CD8^+^ T cells in PBMCs after co-culture. **(G–I)** Flow cytometry analysis of the function of CD8^+^ T cells in PBMCs after co-culture. Data were expressed as means ± SD of three independent experiments. **P* < 0.05, ***P* < 0.01.

Supplementary Figure 3 Hsa_circ_0046523 functions as a sponge of miR-148a-3p. PC cells with downregulated miR-148a-3p or combined with knocked down hsa_circ_0046523 were co-cultured with PBMC cells. **(A–C)** Flow cytometry analysis of the proportion of CD4^+^, CD8^+^ T cells and Tregs in PBMCs after co-culture. **(D–F)** Flow cytometry analysis of apoptosis, exhaustion and function of CD8^+^ T cells in PBMCs after co-culture. **(G, H)** ELISA analysis of the levels of the immune effect cytokines IFN-γ and IL-2, and immunosuppressive cytokines IL-10 and TGF-β in the supernatant of PBMC cells after co-culture. Data were expressed as means ± SD of three independent experiments. **P* < 0.05, ***P* < 0.01, ****P* < 0.001.

Supplementary Figure 4 Downregulation of miR-148a-3p induced the formation of an immunosuppressive microenvironment in PC *via* PD-L1. PC cells with miR-148a-3p knockdown were co-cultured with PBMCs, and PD-L1 neutralizing antibody (anti-PD-L1) was added to the co-culture system. **(A–C)** Flow cytometry analysis of the proportion of CD4^+^, CD8^+^ T cells and Tregs in PBMCs after co-culture. **(D–F)** Flow cytometry analysis of apoptosis, exhaustion and function of CD8^+^ T cells in PBMCs after co-culture. **(G, H)** ELISA analysis of the levels of the immune effect cytokines IFN-γ and IL-2, and immunosuppressive cytokines IL-10 and TGF-β in the supernatant of PBMC cells after co-culture. Data were expressed as means ± SD of three independent experiments. **P* < 0.05, ***P* < 0.01, ****P* < 0.001.

## References

[1] Ferlay J Colombet M Soerjomataram I Mathers C Parkin DM Pineros M. Estimating the Global Cancer Incidence and Mortality in 2018: GLOBOCAN Sources and Methods. Int J Cancer (2019) 144(8):1941–53. doi: 10.1002/ijc.31937 30350310

[2] Siegel RL Miller KD Fuchs HE Jemal A. Cancer Statistics, 2021. CA Cancer J Clin (2021) 71(1):7–33. doi: 10.3322/caac.21654 33433946

[3] Wormann SM Diakopoulos KN Lesina M Algul H. The Immune Network in Pancreatic Cancer Development and Progression. Oncogene (2014) 33(23):2956–67. doi: 10.1038/onc.2013.257 23851493

[4] Melstrom LG Salazar MD Diamond DJ. The Pancreatic Cancer Microenvironment: A True Double Agent. J Surg Oncol (2017) 116(1):7–15. doi: 10.1002/jso.24643 28605029 PMC5989710

[5] Ostrand-Rosenberg S Sinha P Beury DW Clements VK. Cross-Talk Between Myeloid-Derived Suppressor Cells (MDSC), Macrophages, and Dendritic Cells Enhances Tumor-Induced Immune Suppression. Semin Cancer Biol (2012) 22(4):275–81. doi: 10.1016/j.semcancer.2012.01.011 PMC3701942 22313874

[6] Moon YW Hajjar J Hwu P Naing A. Targeting the Indoleamine 2,3-Dioxygenase Pathway in Cancer. J Immunother Cancer (2015) 3:51. doi: 10.1186/s40425-015-0094-9 26674411 PMC4678703

[7] Gajewski T. Innate and Adaptive Immune Cells in Tumor Microenvironment. Gulf J Oncol (2021) 1(35):77–81. 33716216

[8] Boussiotis VA. Molecular and Biochemical Aspects of the PD-1 Checkpoint Pathway. N Engl J Med (2016) 375(18):1767–78. doi: 10.1056/NEJMra1514296 PMC5575761 27806234

[9] Liu J Yang Y Wang H Wang B Zhao K Jiang W. Syntenin1/MDA-9 (SDCBP) Induces Immune Evasion in Triple-Negative Breast Cancer by Upregulating PD-L1. Breast Cancer Res Treat (2018) 171(2):345–57. doi: 10.1007/s10549-018-4833-8 29845474

[10] Zhou WY Zhang MM Liu C Kang Y Wang JO Yang XH. Long Noncoding RNA LINC00473 Drives the Progression of Pancreatic Cancer *via* Upregulating Programmed Death-Ligand 1 by Sponging microRNA-195-5p. J Cell Physiol (2019) 234(12):23176–89. doi: 10.1002/jcp.28884 31206665

[11] Lee A Lim S Oh J Lim J Yang Y Lee MS. NDRG2 Expression in Breast Cancer Cells Downregulates PD-L1 Expression and Restores T Cell Proliferation in Tumor-Coculture. Cancers (Basel) (2021) 13(23):6112. doi: 10.3390/cancers13236112 34885221 PMC8656534

[12] Hayes J Peruzzi PP Lawler S. MicroRNAs in Cancer: Biomarkers, Functions and Therapy. Trends Mol Med (2014) 20(8):460–9. doi: 10.1016/j.molmed.2014.06.005 25027972

[13] Huang Z Wen J Yu J Liao J Liu S Cai N. MicroRNA-148a-3p Inhibits Progression of Hepatocelluar Carcimoma by Repressing SMAD2 Expression in an Ago2 Dependent Manner. J Exp Clin Cancer Res (2020) 39(1):150. doi: 10.1186/s13046-020-01649-0 32746934 PMC7401232

[14] Zocchi L Mehta A Wu SC Wu J Gu Y Wang J. Chromatin Remodeling Protein HELLS is Critical for Retinoblastoma Tumor Initiation and Progression. Oncogenesis (2020) 9(2):25. doi: 10.1038/s41389-020-0210-7 32071286 PMC7028996

[15] Shi L Xi J Xu X Peng B Zhang B. MiR-148a Suppressed Cell Invasion and Migration *via* Targeting WNT10b and Modulating Beta-Catenin Signaling in Cisplatin-Resistant Colorectal Cancer Cells. BioMed Pharmacother (2019) 109:902–9. doi: 10.1016/j.biopha.2018.10.080 30551544

[16] Bao C Guo L. MicroRNA-148a-3p Inhibits Cancer Progression and is a Novel Screening Biomarker for Gastric Cancer. J Clin Lab Anal (2020) 34(10):e23454. doi: 10.1002/jcla.23454 32785967 PMC7595888

[17] Peng L Liu Z Xiao J Tu Y Wan Z Xiong H. MicroRNA-148a Suppresses Epithelial-Mesenchymal Transition and Invasion of Pancreatic Cancer Cells by Targeting Wnt10b and Inhibiting the Wnt/beta-Catenin Signaling Pathway. Oncol Rep (2017) 38(1):301–8. doi: 10.3892/or.2017.5705 28586066

[18] Fu X Hong L Yang Z Tu Y Xin W Zha M. MicroRNA-148a-3p Suppresses Epithelial-to-Mesenchymal Transition and Stemness Properties *via* Wnt1-Mediated Wnt/beta-Catenin Pathway in Pancreatic Cancer. J Cell Mol Med (2020) 24(22):13020–35. doi: 10.1111/jcmm.15900 PMC7701524 33026174

[19] Ashizawa M Okayama H Ishigame T Thar MA Saito K Ujiie D. miRNA-148a-3p Regulates Immunosuppression in DNA Mismatch Repair-Deficient Colorectal Cancer by Targeting PD-L1. Mol Cancer Res (2019) 17(6):1403–13. doi: 10.1158/1541-7786.MCR-18-0831 30872332

[20] Yao T Chen Q Fu L Guo J. Circular RNAs: Biogenesis, Properties, Roles, and Their Relationships With Liver Diseases. Hepatol Res (2017) 47(6):497–504. doi: 10.1111/hepr.12871 28185365

[21] Dong Y He D Peng Z Peng W Shi W Wang J. Circular RNAs in Cancer: An Emerging Key Player. J Hematol Oncol (2017) 10(1):2. doi: 10.1186/s13045-016-0370-2 28049499 PMC5210264

[22] Liu S Li Q Ma Y Corpe C Wang J. Circular RNAs as Novel Potential Biomarkers for Pancreatic Cancer. J Cancer (2021) 12(15):4604–15. doi: 10.7150/jca.58640 PMC8210554 34149924

[23] Li H Hao X Wang H Liu Z He Y Pu M. Circular RNA Expression Profile of Pancreatic Ductal Adenocarcinoma Revealed by Microarray. Cell Physiol Biochem (2016) 40(6):1334–44. doi: 10.1159/000453186 27997903

[24] Guo S Xu X Ouyang Y Wang Y Yang J Yin L. Microarray Expression Profile Analysis of Circular RNAs in Pancreatic Cancer. Mol Med Rep (2018) 17(6):7661–71. doi: 10.3892/mmr.2018.8827 PMC5983963 29620241

[25] Zhao B Li Z Qin C Li T Wang Y Cao H. Mobius Strip in Pancreatic Cancer: Biogenesis, Function and Clinical Significance of Circular RNAs. Cell Mol Life Sci (2021) 78(17-18):6201–13. doi: 10.1007/s00018-021-03908-5 PMC11073466 34342664

[26] Wang Y Yin L Sun X. CircRNA Hsa_Circ_0002577 Accelerates Endometrial Cancer Progression Through Activating IGF1R/PI3K/Akt Pathway. J Exp Clin Cancer Res (2020) 39(1):169. doi: 10.1186/s13046-020-01679-8 32847606 PMC7450704

[27] Huang G Liang M Liu H Huang J Li P Wang C. CircRNA Hsa_circRNA_104348 Promotes Hepatocellular Carcinoma Progression Through Modulating miR-187-3p/RTKN2 Axis and Activating Wnt/beta-Catenin Pathway. Cell Death Dis (2020) 11(12):1065. doi: 10.1038/s41419-020-03276-1 33311442 PMC7734058

[28] Chen S Chen C Hu Y Song G Shen X. The Diverse Roles of Circular RNAs in Pancreatic Cancer. Pharmacol Ther (2021) 226:107869. doi: 10.1016/j.pharmthera.2021.107869 33895187

[29] Wong CH Lou UK Li Y Chan SL Tong JH To KF. CircFOXK2 Promotes Growth and Metastasis of Pancreatic Ductal Adenocarcinoma by Complexing With RNA-Binding Proteins and Sponging MiR-942. Cancer Res (2020) 80(11):2138–49. doi: 10.1158/0008-5472.CAN-19-3268 32217695

[30] Guo X Zhou Q Su D Luo Y Fu Z Huang L. Circular RNA circBFAR Promotes the Progression of Pancreatic Ductal Adenocarcinoma *via* the miR-34b-5p/MET/Akt Axis. Mol Cancer (2020) 19(1):83. doi: 10.1186/s12943-020-01196-4 32375768 PMC7201986

[31] Dougan SK. The Pancreatic Cancer Microenvironment. Cancer J (2017) 23(6):321–5. doi: 10.1097/PPO.0000000000000288 29189327

[32] Wang X Li X Wei X Jiang H Lan C Yang S. PD-L1 is a Direct Target of Cancer-FOXP3 in Pancreatic Ductal Adenocarcinoma (PDAC), and Combined Immunotherapy With Antibodies Against PD-L1 and CCL5 is Effective in the Treatment of PDAC. Signal Transduct Target Ther (2020) 5(1):38. doi: 10.1038/s41392-020-0144-8 32300119 PMC7162990

[33] Zheng H Ning Y Zhan Y Liu S Yang Y Wen Q. Co-Expression of PD-L1 and HIF-1alpha Predicts Poor Prognosis in Patients With Non-Small Cell Lung Cancer After Surgery. J Cancer (2021) 12(7):2065–72. doi: 10.7150/jca.53119 PMC7974520 33754005

[34] Francisco LM Salinas VH Brown KE Vanguri VK Freeman GJ Kuchroo VK. PD-L1 Regulates the Development, Maintenance, and Function of Induced Regulatory T Cells. J Exp Med (2009) 206(13):3015–29. doi: 10.1084/jem.20090847 PMC2806460 20008522

[35] Que Y Xiao W Guan YX Liang Y Yan SM Chen HY. PD-L1 Expression Is Associated With FOXP3+ Regulatory T-Cell Infiltration of Soft Tissue Sarcoma and Poor Patient Prognosis. J Cancer (2017) 8(11):2018–25. doi: 10.7150/jca.18683 PMC5559963 28819402

[36] Chen JX Yi XJ Gao SX Sun JX. The Possible Regulatory Effect of the PD-1/PD-L1 Signaling Pathway on Tregs in Ovarian Cancer. Gen Physiol Biophys (2020) 39(4):319–30. doi: 10.4149/gpb_2020011 32902402

