# Supplementary figures and images for: Hsa_circ_0046523 Mediates an Immunosuppressive Tumor Microenvironment by Regulating MiR-148a-3p/PD-L1 Axis in Pancreatic Cancer
Source: Front Oncol. 2022 May 30;12:877376. doi: 10.3389/fonc.2022.877376 (PMC9192335; doi:10.3389/fonc.2022.877376)

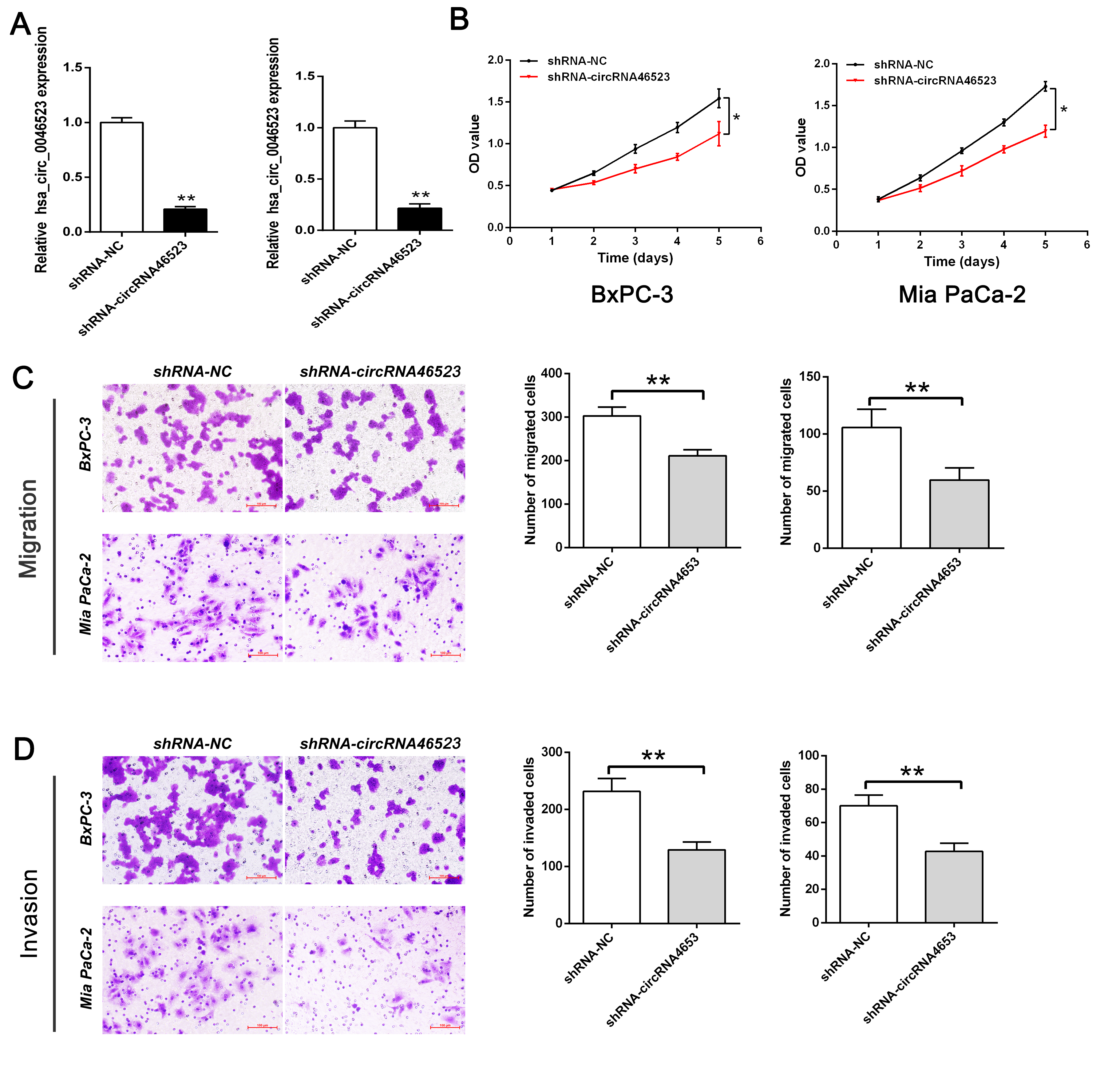

Supplement: Supplementary Figure 1 — Hsa_circ_0046523 knockdown inhibited the proliferation, migration and invasion of PC cells. (A) After infecting lentivirus encoding sh-circRNA46523, the expression level of hsa_circ_0046523 in BxPC-3 and Mia PaCa-2 cells was confirmed using RT-qPCR analysis. (B) CCK‐8 assays were conducted to estimate the effects of hsa_circ_0046523 knockdown on the proliferation of BxPC-3 and Mia PaCa-2 cells. (C, D) Transwell assays were conducted to estimate the effects of hsa_circ_0046523 knockdown on the cell migration and invasion capacities of BxPC-3 and Mia PaCa-2 cells. Data were expressed as means ± SD of three independent experiments. *P < 0.05, **P < 0.01. [file Image_1.tif]

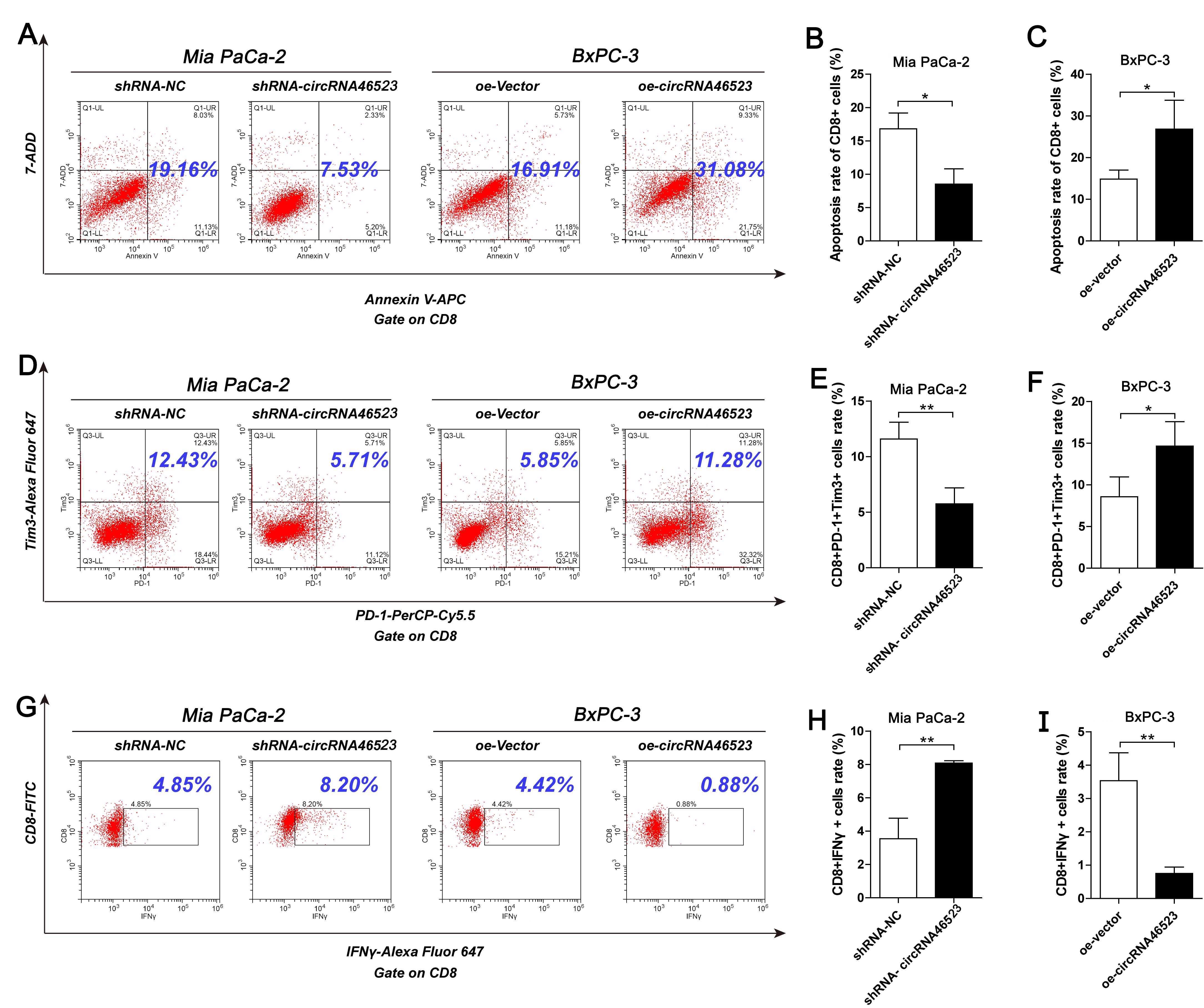

Supplement: Supplementary Figure 2 — Hsa_circ_0046523 regulated the apoptosis, exhaustion and function of CD8+ T cells in the co-cultured system. PC cells overexpressing or knocking down hsa_circ_0046523 were co-cultured with PBMC cells. (A–C) Flow cytometry analysis of the apoptosis of CD8+ T cells in PBMCs after co-culture. (D–F) Flow cytometry analysis of the exhaustion of CD8+ T cells in PBMCs after co-culture. (G–I) Flow cytometry analysis of the function of CD8+ T cells in PBMCs after co-culture. Data were expressed as means ± SD of three independent experiments. *P < 0.05, **P < 0.01. [file Image_2.tif]

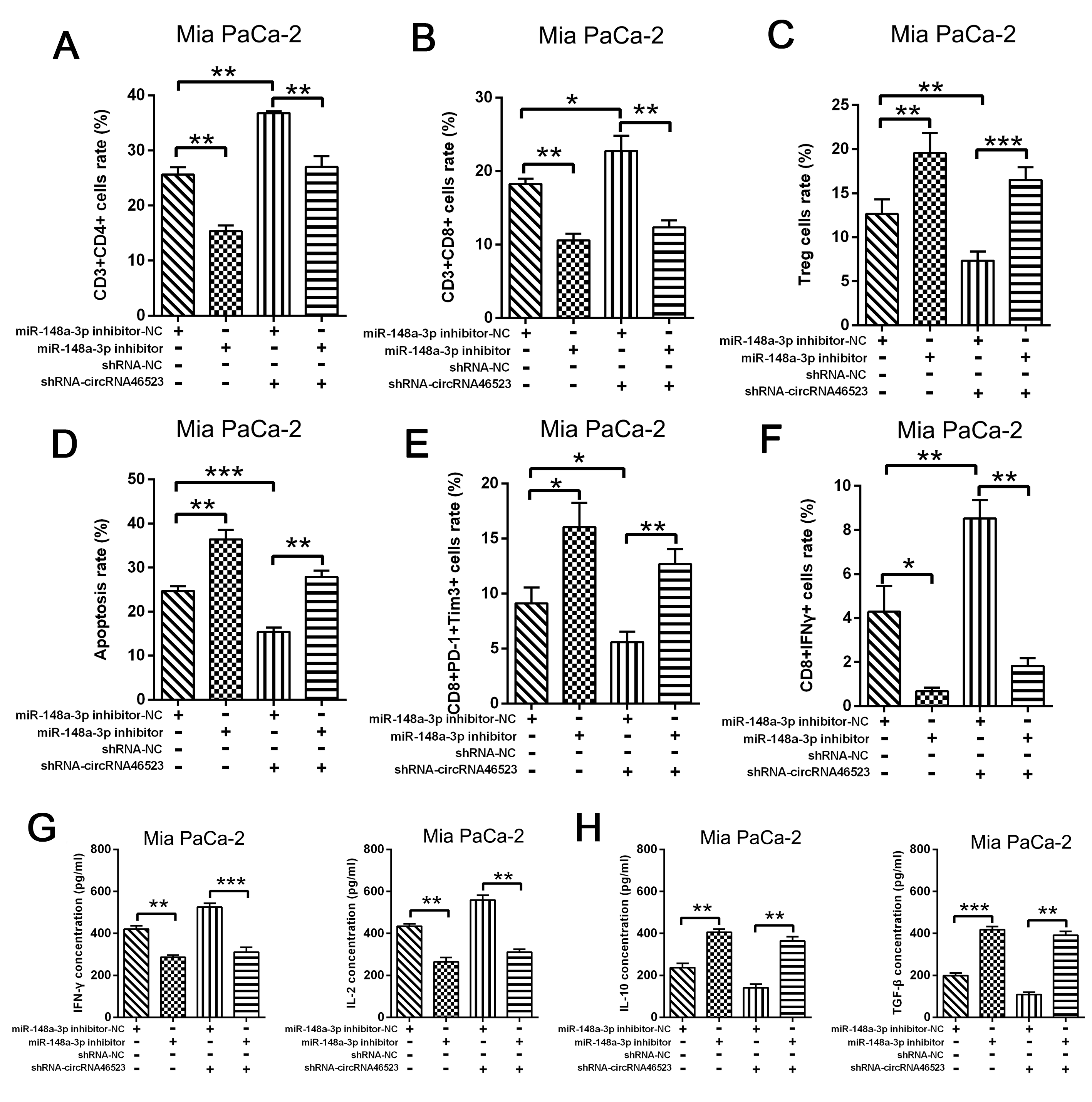

Supplement: Supplementary Figure 3 — Hsa_circ_0046523 functions as a sponge of miR-148a-3p. PC cells with downregulated miR-148a-3p or combined with knocked down hsa_circ_0046523 were co-cultured with PBMC cells. (A–C) Flow cytometry analysis of the proportion of CD4+, CD8+ T cells and Tregs in PBMCs after co-culture. (D–F) Flow cytometry analysis of apoptosis, exhaustion and function of CD8+ T cells in PBMCs after co-culture. (G, H) ELISA analysis of the levels of the immune effect cytokines IFN-γ and IL-2, and immunosuppressive cytokines IL-10 and TGF-β in the supernatant of PBMC cells after co-culture. Data were expressed as means ± SD of three independent experiments. *P < 0.05, **P < 0.01, ***P < 0.001. [file Image_3.tif]

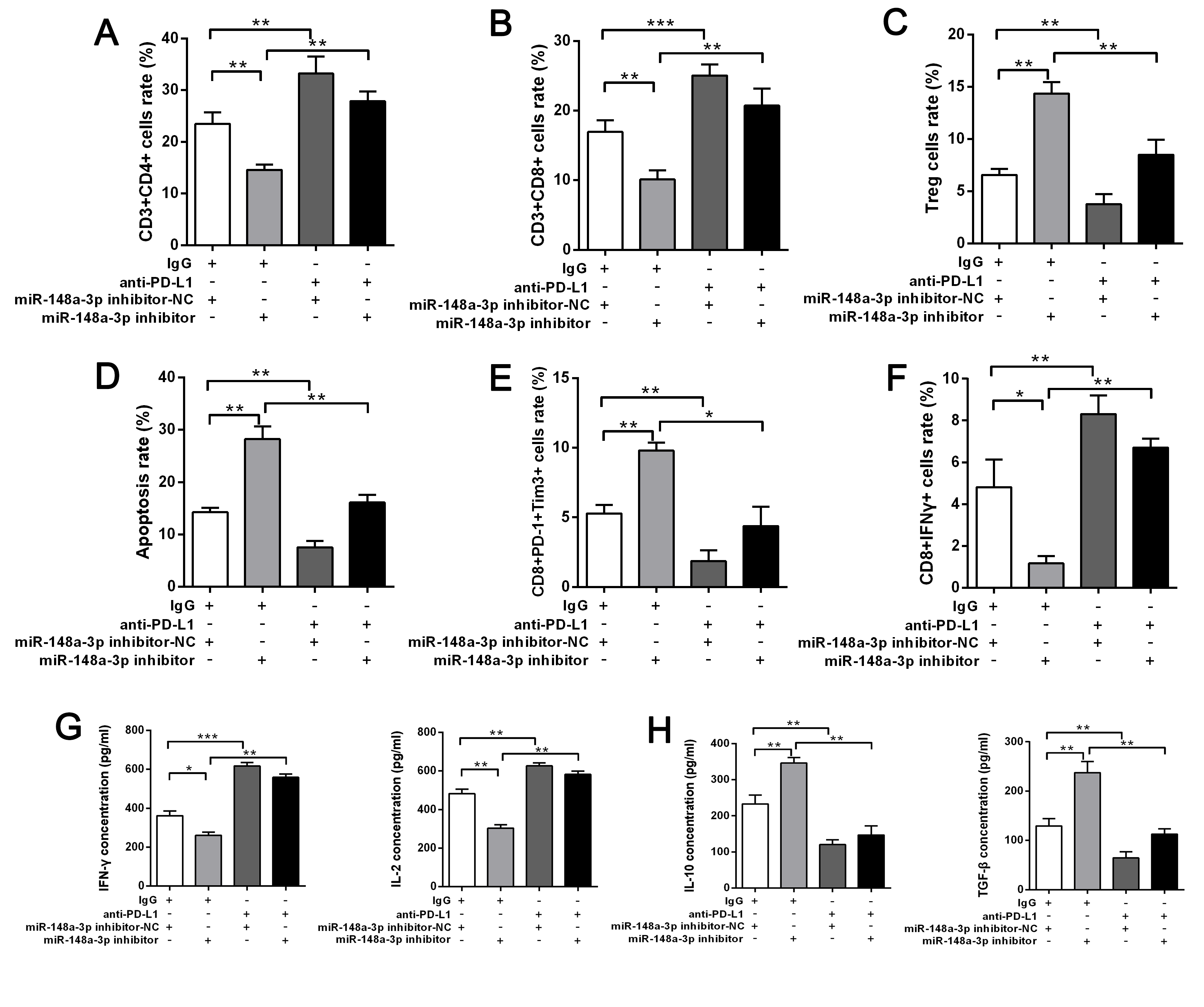

Supplement: Supplementary Figure 4 — Downregulation of miR-148a-3p induced the formation of an immunosuppressive microenvironment in PC via PD-L1. PC cells with miR-148a-3p knockdown were co-cultured with PBMCs, and PD-L1 neutralizing antibody (anti-PD-L1) was added to the co-culture system. (A–C) Flow cytometry analysis of the proportion of CD4+, CD8+ T cells and Tregs in PBMCs after co-culture. (D–F) Flow cytometry analysis of apoptosis, exhaustion and function of CD8+ T cells in PBMCs after co-culture. (G, H) ELISA analysis of the levels of the immune effect cytokines IFN-γ and IL-2, and immunosuppressive cytokines IL-10 and TGF-β in the supernatant of PBMC cells after co-culture. Data were expressed as means ± SD of three independent experiments. *P < 0.05, **P < 0.01, ***P < 0.001. [file Image_4.tif]
