# Supplementary material for: Hsa_circ_0046523 Mediates an Immunosuppressive Tumor Microenvironment by Regulating MiR-148a-3p/PD-L1 Axis in Pancreatic Cancer
Source: Front Oncol. 2022 May 30;12:877376. doi: 10.3389/fonc.2022.877376 (PMC9192335; doi:10.3389/fonc.2022.877376)
Supplement: Supplementary file 5 [file Table_3.doc]

**TABLE S1 | qRT-PCR primers used in this study.**

| Genes | Primers | Sequences (5'-3') |
| --- | --- | --- |
| *hsa_circ_0046523* | Forward | CGCAGCCAATGATAGACCAC |
|  | Reverse | GATCACGGCGACAACCATC |
| *hsa-miR-148a-3p* | Forward | GAGGTCAGGGTCAGTGCACTACAGAAC |
|  | Reverse | CTCAAGTGTCGTGGAGTCGGCAA |
| *mmu-miR-148a-3p* | Forward | AGCAGTTCAGTGCACTACAG |
|  | Reverse | AGCAGTTCAGTGCACTACAG |
| *Human PD-L1* | Forward | CCTACTGGCATTTGCTGAACGCAT |
|  | Reverse | ACCATAGCTGATCATGCAGCGGTA |
| *Mouse PD-L1* | Forward | ATGAGGATATTTGCTGGCATTA |
|  | Reverse | TTACGTCTCCTCGAATTGTGT |
| *U6* | Forward | CTCGCTTCGGCAGCACA |
|  | Reverse | AACGCTTCACGAATTTGCGT |
| *GAPDH* | Forward | GCACCGTCAAGGCTGAGAAC |
|  | Reverse | TGGTGAAGACGCCAGTGGA |
