# Supplementary material for: Hsa_circ_0046523 Mediates an Immunosuppressive Tumor Microenvironment by Regulating MiR-148a-3p/PD-L1 Axis in Pancreatic Cancer
Source: Front Oncol. 2022 May 30;12:877376. doi: 10.3389/fonc.2022.877376 (PMC9192335; doi:10.3389/fonc.2022.877376)
Supplement: Supplementary file 6 [file Table_4.doc]

**TABLE S2 | Flow cytometry antibodies used in this study.**

| Antibody | Souse | Catalog no. |
| --- | --- | --- |
| *Anti-human CD3-PE* | BD Biosciences | 555340 |
| *Anti-human CD4-FITC*  *Anti-human CD8-FITC* | BD Biosciences  BD Biosciences | 555346  557085 |
| *Anti-human CD25-PerCP-Cy5*  *Anti-human Foxp3-Alexa Fluor 648*  *Anti-human PD-1-PerCP-Cy5.5*  *Anti-human Tim3-Alexa Fluor 647*  *Anti-human PD-L1-FITC*  *Anti-mouse CD3-PE*  *Anti-mouse CD4-FITC*  *Anti-mouse CD8-FITC*  *Anti-mouse CD25-PerCP-Cy5.5*  *Anti-mouse Foxp3-Alexa Fluor 647*  *Anti-mouse IFNγ-Alexa Fluor 647*  *Anti-mouse PD-1-Alexa Fluor647*  *Anti-mouse Tim3-PerCP-Cy5.5* | BD Biosciences  BD Biosciences  BD Biosciences  BD Biosciences  BD Biosciences  BD Biosciences  BD Biosciences  BD Biosciences  BD Biosciences  BD Biosciences  BD Biosciences  BD Biosciences  BD Biosciences | 555433  561184  561273  565559  558065  553063  553650  553030  563598  563486  557735  566715  567123 |
